# Long-term Remission Over Six Years for a Patient with Recurrent Glioblastoma Treated with Cediranib/Lomustine

**DOI:** 10.7759/cureus.460

**Published:** 2016-01-16

**Authors:** Doniel Drazin, Lutfi Al-Khouja, Ashish Patel, Jethro Hu, Surasak Phuphanich

**Affiliations:** 1 Neurosurgery, Cedars-Sinai Medical Center; 2 Surgery, UCSD School of Medicine; 3 Neurology, Cedars-Sinai Medical Center

**Keywords:** Neurosurgery, glioblastoma multiforme, recurrent glioblastoma, brain tumor

## Abstract

Cediranib is an orally available, pan-VEGFR tyrosine kinase inhibitor. A previous Phase III study of patients with recurrent glioblastoma treated with this drug did not meet the primary end of progressive-free survival (PFS). We identified one patient, a 57-year-old Caucasian female who, following surgery in October 2008 and concurrent temozolomide and radiation therapy from November 8, 2008, to January 6, 2009, developed a tumor progression of the left posterior frontal measuring 1.2 x 1.5 cm in February 2009. She was enrolled in a randomized, Phase III, placebo-controlled, partially-blinded clinical trial of cediranib as either monotherapy or in combination with lomustine (CCNU) versus CCNU. She was randomized to receive a combination therapy with 1st cycle CCNU 190 mg and cediranib 20 mg per day on April 15, 2009. However, she developed nephrotic syndrome and uncontrolled hypertension and was taken off this study in May 2010. Her six-week MRI showed a 50% tumor regression and a complete response at twenty-four weeks. With no enhancement seen on MRI on June 4, 2015, she has been off therapy and in clinical remission over five years with high functional level and good quality of life (KPS-90%). This is a case report of successful therapy for recurrent glioblastoma with long-term remission despite termination of therapy greater than six years from cediranib and limited CCNU dosage.

## Introduction

Glioblastoma multiforme (GBM) is the most common, aggressive, primary brain tumor in adults with a relatively poor prognosis. There are an estimated 10,000 cases annually in the United States with a median survival of 14.6 months and a five-year survival rate of 5%. Almost all patients with GBM eventually relapse after treatment [1].

GBM has been characteristically shown to express high levels of pro-angiogenic cytokines leaving a potential target area of pharmacologic therapy to prevent growth of these tumors [2]. Anti-VEGF and anti-VEGFR agents have been in the forefront of research in GBM therapies over the past decade and yet there is still much to be explored on the effects of these regimens on primary and recurrent GBM. Bevacizumab is currently the most widely used agent for recurrent GBM. Originally, it showed to have high response rates and six-month progression-free survival (PFS), but later studies that demonstrated no survival benefit with the addition of bevacizumab made its utility much more unclear in the treatment of recurrent GBM. More recently, the Phase II BELOB trial from the Netherlands demonstrated that bevacizumab in monotherapy does not play a significant role in recurrent GBM treatment and should not proceed to Phase III trial [3]. In light of this, more definitive research to find an effective treatment has become more important.

Several clinical trials have been performed to determine the effectiveness of cediranib on recurrent GBM. Cediranib is an orally available, pan-VEGFR tyrosine kinase inhibitor, unlike bevacizumab which is a VEGF-A inhibitor. While bevacizumab prevents the interaction between VEGF and its receptor on endothelial cells preventing proliferation and angiogenesis, it is more specifically limited to VEGF-A, which is not a ligand for the VEGF receptor 3 (VEGFR-3). VEGFR-3 is more specifically known for its role in lymphangiogenesis. Cediranib targets the VEGF receptors instead of the ligands and has shown to have activity against VEGFR-1, -2, and -3, which provides broader inhibition of the VEGF pathway and, theoretically, a more effective modality to halt angiogenesis. Most of the prior research on cediranib was involved in its effectiveness in gynecologic cancer in the ICON6 trial, which demonstrated improvement in both PFS and overall survival (OS) [4]. This trial showed the OS was limited to 2.7 months. Although most of the prior research studied the use of cediranib in gynecologic cancers in the past, it is within the same drug class as bevacizumab, both being VEGF-signaling inhibitors. While bevacizumab is currently the standard therapy for the treatment of GBM, cediranib theoretically should also have some efficacy on patients with these tumors given its similar mechanism of action. However, a previous Phase III study in patients with recurrent glioblastoma, cediranib did not meet primary end of PFS in monotherapy or in combination with lomustine [5]. Another study of cediranib and cilengitide did not have promising survival and response rates, further demonstrating the lack of effectiveness of cediranib therapy in recurrent GBM [6]. However, we found one patient from this study,  a 57-year-old Caucasian female, who developed tumor progression of the left posterior frontal region four months after primary surgical resection of her tumor and has had greater than six years of remission after undergoing cediranib and limited-dose CCNU therapy. 

## Case presentation

### History

**Figure 1:**
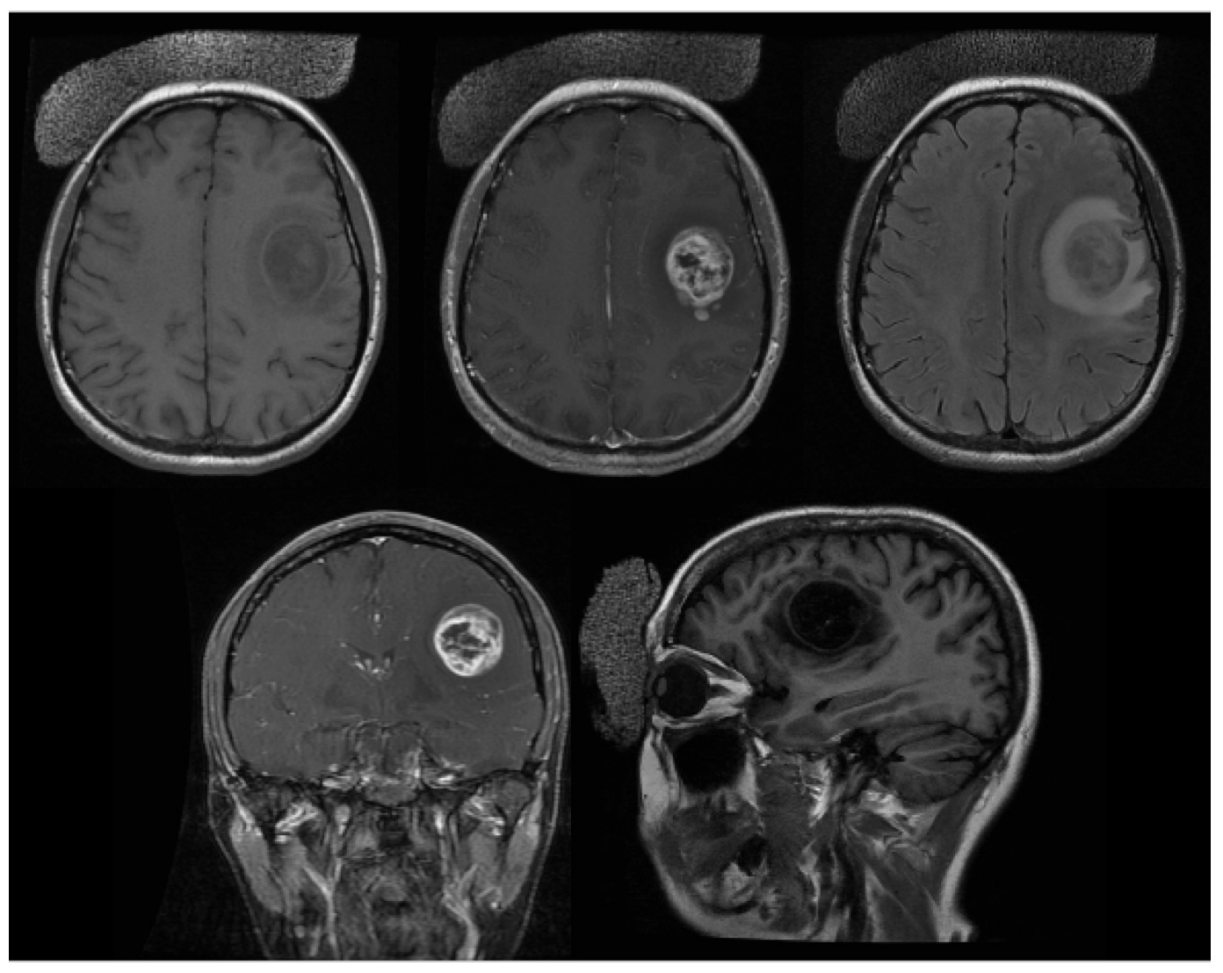
Preoperative Brain MRI Brain MRI shows left frontotemporal mass and T2/FLAIR hyperintensity

**Figure 2:**
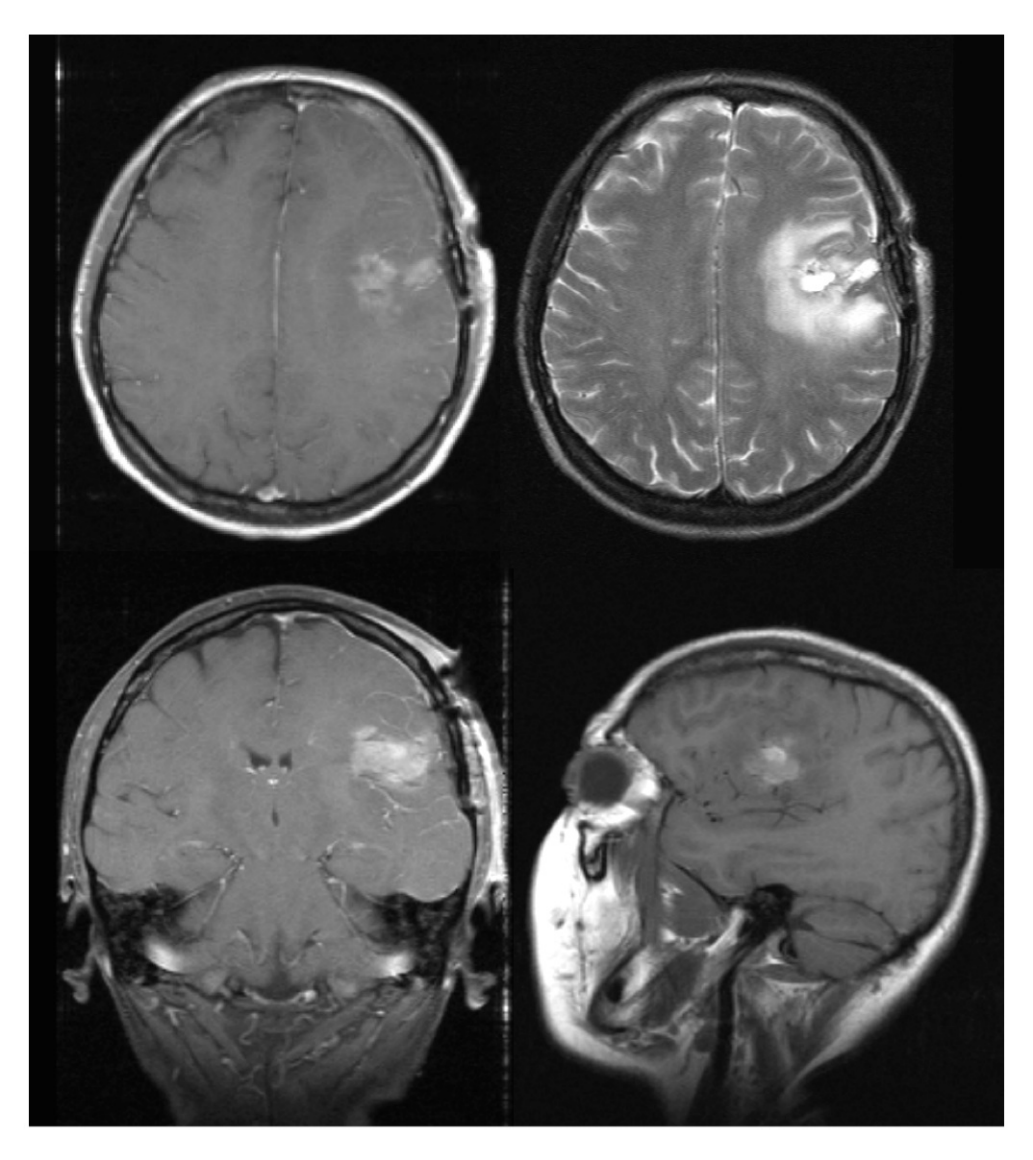
Postoperative Brain MRI Brain MRI after tumor excision showing post-operative changes in the left frontotemporal region

This is a 57-year-old, right-handed, Caucasian female who originally presented to another institution after an episode of loss of consciousness with postictal expressive aphasia. At the time, MRI of the brain showed a left frontotemporal mass (Figure 1), and she subsequently underwent a left temporal craniotomy with total resection of the tumor on October 15, 2008 (Figure 2).

### Pathology

The pathology report provided a diagnosis of Glioblastoma multiforme, WHO Grade IV.  Microscopic evaluation featured moderately cellular proliferation of highly atypical astrocytes with enlarged hyperchromatic nuclei associated with fibrillary cytoplasm and common gemistocytic cells. There were numerous mitotic figures. Endothelial proliferation was detected and some blood vessels were cuffed by mature lymphocytes. Myxoid degeneration with prominence of blood vessels was seen with multiple layers of necrosis. Immunostaining showed pMAPK positivity in 60% of tumor cell cytoplasm and 30% of tumor nuclei.  There was loss of PTEN with staining in less than 20% of tumor cells. MGMT was positive in less than 1% of tumor cells (methylated MGMT).

### Postoperative course

The patient underwent radiation and concurrent temozolomide 140 mg from November 8, 2008, to January 6, 2009, which was complicated by thrombocytopenia with bleeding requiring transfusion and missing the last four days of treatment. A follow-up MRI scan from February 18, 2009, demonstrated an increased satellite lesion in the left temporal region. She was given the recommendation for stereotactic radiosurgery or participation with a Phase III clinical trial of cediranib versus cediranib and CCNU. The patient was then enrolled in this clinical trial, and she was randomized to receive a combination of cediranib 20 mg and CCNU 190 mg on April 15, 2009. Four weeks later, she developed Grade 4 thrombocytopenia associated with gum bleeding and dizziness necessitating a platelet transfusion. MRI at the time showed decreased enhancement and less edema on T2/FLAIR in the left frontal region indicating tumor size reduction compared to the March 30, 2009, scan. Given her persistent thrombocytopenia, CCNU was held and she continued monotherapy with cediranib at 20 mg per day. She had a second, reduced dose of CCNU of 100 mg on June 17, 2009, and was later hospitalized from July 24 - 27, 2009, for generalized tonic-clonic seizures. She was restarted on keppra and discharged home. MRI scan on July 27, 2009, during her hospitalization was stable compared to prior images and showed a mild residual enhancement around the left frontal resection cavity and stable T2/FLAIR hyperintensity. She was again hospitalized on January 28, 2010, for severe-range hypertension, proteinuria, and diarrhea, and was discharged home after her blood pressure improved. She was continued on cediranib and did not receive the third dose of CCNU. In total, she received two doses of CCNU (190 mg on April 13, 2009, and 100 mg on June 17, 2009). The cediranib dose was reduced to 15 mg per day starting March 16, 2010, and was eventually held on May 8, 2010, resulting in improvement in the patient’s appetite and fatigue. During therapy, she required five anti-hypertensive medications from the side effects of cediranib. The patient was subsequently discontinued from participating in the trial on May 20, 2010, because of persistent Grade 3 proteinuria. At her six-week follow-up, the MRI showed 50% tumor regression and complete response at week 24 of the study. With no enhancement having been seen on the MRI on September 3, 2015, she has been off therapy and in clinical remission for over six years with high functional level and good quality of life (KPS 90%) with only one medication for hypertension.

## Discussion

This is a case report of successful therapy for recurrent GBM with long-term remission despite termination of therapy greater than six years from cediranib and limited CCNU dosage that is contrary to the results currently published [5-6]. Since discontinuation from the clinical trial greater than five years ago, multiple MRIs showed stable disease, and the patient has a KPS of 90%. Looking back at Batchelor, et al.’s randomized Phase III clinical trial of cediranib in monotherapy versus combination therapy with lomustine versus lomustine in monotherapy in recurrent GBM, the authors concluded that cediranib showed evidence of clinical activity regarding time to deterioration in neurological status and corticosteroid-sparing effects [5]. However, this study did not meet the primary end point of PFS in any of the research arms. The side-effect profile of the patient presented was not uncommon in Batchelor, et al.’s clinical trial with 38.3% of patients who received combination therapy experienced thrombocytopenia and 79.7% had adverse events during therapy [5].

The reason the patient in this case report did not meet the primary end point may be due to multiple reasons including specific tumor properties making it less susceptible to the therapy and the lower dose of treatment than the clinical trial set. Similar to how not all GBM tumors respond to bevacizumab, which is currently the standard therapy, not all GBM tumors may respond to cediranib. Therefore, it is hard to designate one standard therapy regimen to treat all GBMs as some will respond better to certain therapies than others. The patient presented here showed great response to this therapy having received a successful yet unusual outcome from combination cediranib and lower-dosed CCNU therapy, which has not been yet described in the treatment of GBM.

The utility of anti-VEGF agents such as cediranib are still being explored in mono- and combination therapies, with no current studies showing impressive results in terms of PFS and OS. A recently published Phase I study of cediranib and cilengitide, another anti-angiogenic agent targeting integrins, did not show any clinical benefit [6]. Additionally, their findings suggest that combination therapy of cediranib and cilengitide may have an antagonistic effect given no statistically significant decrease in Ang2 levels, as what would be expected in monotherapy with either drug. This further calls for research to better elucidate the effects of these therapies on VEGF and resistance mechanisms that may be encountered during treatment for recurrence. 

## Conclusions

This case report highlights a successful and unusual outcome from the combination of cediranib and CCNU therapy. Although the current literature did not show overall benefit from this combination, the impressive outcome obtained in this case only demonstrated the benefit of cediranib in this patient.
